# Nanomaterials for the Treatment of Heavy Metal Contaminated Water

**DOI:** 10.3390/polym14030583

**Published:** 2022-01-31

**Authors:** Rabia Baby, Mohd Zobir Hussein, Abdul Halim Abdullah, Zulkarnain Zainal

**Affiliations:** 1 Nanomaterial Synthesis and Characterization Laboratory, Institute of Nanoscience and Nanotechnology (ION2), Universiti Putra Malaysia, Serdang 43400, Malaysia; rabia.shaikh@iba-suk.edu.pk; 2 Department of Education, Sukkur IBA University, Sukkur Sindh 65200, Pakistan; 3 Department of Chemistry, Universiti Putra Malaysia, Serdang 43400, Malaysia; halim@upm.edu.my (A.H.A.); zulkar@upm.edu.my (Z.Z.)

**Keywords:** water treatment, layered double hydroxides, iron oxide nanoparticles, metal oxides, nanocomposite

## Abstract

Nanotechnology finds its application almost in every field of science and technology. At the same time, it also helps to find the solution to various environment-related problems, especially water contamination. Nanomaterials have many advantages over conventional materials, such as high surface area, both polar and non-polar chemistries, controlled and size-tunable, easier biodegradation, which made them ideal candidates for water and environmental remediation as well. Herein, applications of non-carbon nanomaterials, such as layered double hydroxides, iron oxide magnetite nanoparticles, nano-polymer composites, metal oxide nanomaterials and nanomembranes/fibers in heavy metal contaminated water and environmental remediation are reviewed. These non-carbon nanomaterials, due to their tunable unique chemistry and small size have greater potentials for water and environmental remediation applications.

## 1. Introduction

**Figure 1:**
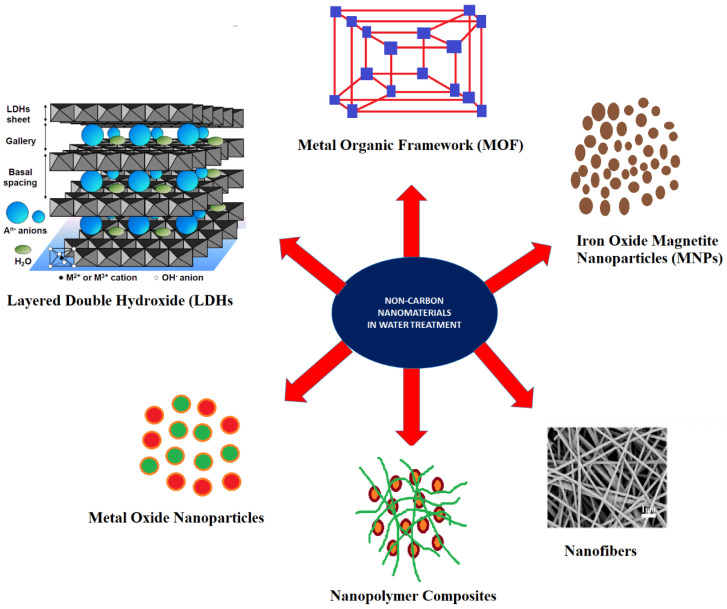
Different non-carbon nanomaterials, used for the treatment of heavy metal-contaminated water.

Pollution is defined as the presence of undesirable substances harming living things and the environment [1,2,3,4,5]. Industrialization and monstrous increment in the populace, prompting developing urbanization causing the expansion in contamination at a disturbing rate [6,7,8,9]. The unadulterated and clean water is getting scarce because of industrialization, and the world is confronting a deficiency of clean water [6,7,10,11,12,13]. Water contaminants can be organics, microbes, dyes, metal particles; lead, cadmium, zinc, nickel, arsenic, chromium and mercury with non-biodegradable nature representing an extraordinary hazard to human wellbeing. Substantial metal particles can cause numerous unfavorable influences like a malignant growth, kidney damage, hepatitis, unsuccessful labor, iron deficiency, encephalopathy and nephritic disorder, and so forth [1,2,6,7,10,11,14]. Nickel particles can cause skin diseases when reached with gems destroying zips, watches, coins and so on. Chromium metal particles (VI) cause maladies like liver harm, nephritis, stomach bothers, etc., Cr (VI) particles are likewise the significant reason for nasal mucous ulcers [15]. Lead (Pb) particles are discharged in nature, mostly from metal mining activities of corrosive lead batteries, paper, glass and cleaning ventures. Cadmium is commonly found in water, released from electroplating of batteries, photovoltaic cells, metallurgy procedures, and textiles industrial facilities [10,16]. Figure 1 shows various sources of heavy metal causing environmental and water pollution and Table 1 shows various diseases caused by metal contamination [2].

Given serious antagonistic health and environmental impacts, the expulsion of metal particles from water is of prime importance for sparing living creatures, especially humans from such dangerous medical problems. The treatment of natural toxins and their anticipation is a key advance in the insurance of good wellbeing. Material science assumes a crucial job in the toxins-free environment and innovation has advanced exponentially in the last decade especially in nanotechnology [2]. Various techniques are being applied for the purification of water, such as filtration, coagulation, biosorption, precipitation, photo-catalysis and extraction, etc., [2,6,17,18,19,20]. Adsorption is considered the best strategy as it is easy, profoundly proficient, and simple to work for expelling exceed maximum levels of metals [1,6,21]. Various materials have been applied for the removal of these contaminants from water, for example by bio-sorbents of agricultural waste, carbon-based materials, such as charcoal, activated carbon, graphene, graphene oxide, single-wall carbon nanotubes, multi-walled carbon nanotubes, fullerenes and various polymeric materials [2,3,22,23]. In addition to this, non-carbon nanomaterials are also widely used in water purification and environmental remediation namely iron oxide magnetite nanoparticles, layered double hydroxides (LDH), metal oxides (MO), metal-organic frameworks (MOF), etc., [24,25]. The carbon nanomaterials e.g., Carbon nanotubes, graphene, graphite, fullerenes and activated carbon are not reviewed here, as we have reviewed them previously [2]. Dawn et al. 2021 have recently briefly reviewed wastewater treatment by the application of agricultural waste and some of the nanoadsorbents [26]. Here we reviewed the recent advancement in the applications of these non-carbon nanomaterials, eg., Nanopolymeric membranes, LDHs, Iron Oxide Magnetite NPs (Fe_3_O_4_), metal-organic framework, for the treatment of heavy metal contaminated water and environmental remediation. This review article will update the scientists and researchers regarding the latest advancement in the fabrication of these nanomaterials for making these materials suitable for their application in the treatment of heavy metals contaminated water.

## 2. Heavy Metal Toxicity

The term toxic metals is described as the elements toxic to living things and most of these metals have a higher atomic weight that is why they are also referred to as heavy metals. Example of heavy metals are Cr (VI), Pb^2+^, Cd^2+^, Mn^2+^, Hg^+^, As^3+^ and radio elements [1,2,6]. Usually, heavy metals are thought of as toxic to living creatures, however, certain lightweight metals are also toxic, such as lithium and beryllium. There are certain heavy metals essential for humans and are non-toxic at a certain amount, such as Cr^3+^ and Fe^+3^ and Fe^2+^, etc. The extent of metal toxicity depends on the route of exposure, time of exposure, dose/quantity, where all these factors contributed to the resulting in acute or chronic toxicity. Chromium (Cr) is found in different oxidation states, however, +3 and +6 are the most stable forms. Chromium in the +3 form is essential for humans because of its different nutritional and biological characteristics [15,27]. However, Cr (VI), is extremely toxic and carcinogenic [27]. World Health Organization (WHO) has set a 0.05 mg/L limit of Cr (VI), in the ground and surface water [28]. Mercury (Hg) is hazardous to the environment and living creatures. It comes to the environment mainly from different industries namely the paper and pulp industry, pharmaceuticals, and agricultural industry, etc., [29,30]. Mercury is the most toxic heavy metal to the environment, the higher level of Hg in any form can cause acrodynia, can be toxic to the brain, kidneys, fetus, lungs and skin [29,31]. In addition to this, mercuric chloride and methyl mercury are declared carcinogenic by the Environmental Protection Agency (EPA). WHO has set 0.01 mg/L as the lower limit in drinking water. Lead (Pb^2+^) is also toxic heavy metal that can cause cancer, mental disorders, allergies, autism, dyslexia and kidney failure, etc., [28,32]. It is mainly produced in the environment from batteries, pesticides, fertilizers, metal plating and smelting of ores, etc. Arsenic (As^3+^) is a toxic heavy metal that can destroy cells by disturbing protoplasm, which can cause respiration malfunctioning and liver damage, etc., [33,34,35]. Arsenic mainly contaminates water and enters the environment from arsenic-based pesticides, or in groundwater from natural mineral deposits and inappropriately disposal of the reagents and chemicals containing arsenic [29,36]. Cadmium is another element that falls in the category of toxic heavy metals having chemical properties similar to Hg [37]. It can cause damage to the lungs, kidneys, skeleton and is also carcinogenic to humans [37,38,39]. It is produced in the environment from plasticizers, pigments, alloys, smelting, nuclear industry and batteries [10,29,30,38]. Zinc is essential for many biological reactions/processes in living things, however, excessive amounts of zinc can cause an absorption decrease of iron which is essential to humans [40,41]. Zinc is very toxic to fish, plants and invertebrates [40,42]. The heavy metals or dissolved hazardous chemicals in water cause many defects not only to humans but also in the wild and aquatic ecosystems [43,44,45]. This is a big challenge for the whole of the world to stop heavy metal pollution to water and the environment.

**Table 1:** The sources, toxicity and WHO permissible limit of heavy metal ions.

Heavy Metal Ions	Sources	Toxicity	WHO Max Limit (mg/L)	References
Cr (VI),	Metallurgy, mining, leather industries, Ferroalloys, etc.	Carcinogenic, stomach disease, puking, and Migraine,	0.05	[27,29,46,47]
Pb^2+^	Batteries, pesticides, fertilizers, metal plating and smelting of ores	Cancer, mental disorders, allergies, autism, dyslexia and kidney failure	0.1	[32,46,48,49]
Cd^2+^	Metal coating batteries, coal burning and pigments	Kidney diseases, Renal problems and carcinogen	0.01	[16,46,48,49,50,51]
As	Pesticides, ceramics, animal supplements, metallurgy, electrical production, geochemical and coals.	Skin disease, Liver diseases, breathing problems and vascular complications	0.05	[10,30,33,36,46]
Hg^1+^	Metallurgy, catalyst, mercury lamps, paper and pulp industry, pharmaceuticals, and agricultural industry, etc.	The nervous system, blood circulatory disorder and kidney failure	0.00003	[29,30,46,52,53]
Cu^2+^	Pharmaceutical and chemical industries, water pipelines and alloys	Liver diseases, and brain	0.25	[2,46,48]
Ni^2+^	Ceramics, glass batteries and catalyst	Lung diseases, a carcinogen and causes skin diseases	0.2	[2,46,48]
Zn^2+^	Zinc alloys, PVC stabilizers, stabilizer, rubber and paint industry	Toxic to aqueous species, to human causes anxiety and lethargy	0.8	[38,42,46,54]

## 3. Layered Double Hydroxides

**Figure 2:**
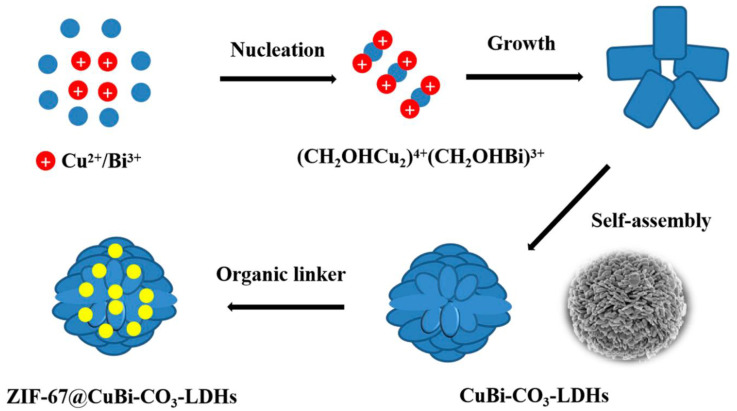
Schematic illustration for the formation of flower-like ZIF-67/CuBi–CO_3_-LDH, reproduced from [60] with permission, copyright The Materials Research Society, 2020.

Layered double hydroxides (LDHs) have recently emerged as a useful material with a variety of applications in drug delivery, bio-imaging, biosensors, chemo-sensors, etc., because of their bio-degradable and biocompatible nature. Most recently, LDHs have been widely applied in water purification and environmental remediation [55,56,57]. Layered magnesium hydroxides or brucite has a chemical formula, [Mg(OH)_6_], where the divalent Mg^2+^ ions are surrounded by six OH^−^ ions in an octahedral manner. These [Mg (OH)_6_] form an infinite layered structure by sharing their edges, and these units are repeated continuously. LDHs are prepared by introducing trivalent cations (Al^3+^, Gd^3+^ and Fe^3+^, etc.) in their structure, resulting in a positive charge in the layered structure balanced by counter anions e.g., NO_3_^−^, CO_3_, organic anions and drugs, etc., [55]. The major problem in industrial wastewater treatment is that it contains both cations and anions, therefore, two different adsorbents with different chemistries are required to treat it. Nano-adsorbents were prepared using Mg/Al-layered double hydroxides-biochar (Mg/A-LDH-BC) derived from sawdust. This was successfully applied as the adsorbent for the treatment of industrial electroplating wastewater containing Pb^2+^ cations and CrO_4_^2−^ anions. The designed adsorbent worked well for the simultaneous adsorption of both cation Pb^2+^ and anions CrO4^2−^. The designed adsorbent was found to have an adsorption capacity of 591.2 mg/g and 330.8 mg/g for Pb^2+^ and anions CrO_4_^2−^, respectively. Moreover, these adsorption capacities were about 263% and 416% higher than the biochar alone [43]. Langmuir was found to be better than the Freundlich isotherm for both ions [43]. Mg/Fe-LDHs-based hollow nano-spheres were prepared using a one-step thermal method where Mg/Fe-LDHs were prepared first followed by heating at 400 °C, resulting in their oxides, Mg/Fe-LDO with maintained nano-hollow structure [44]. The hollow nano-spheres, Mg/Fe-LDO have shown excellent efficiency for the adsorption of As^5+^ and Cr (VI), with 99% removal efficiency in a short time of 5 min. The removal capacity was found to be 178.6 mg g^−1^ and 148.7 mg g^−1^ for As^5+^ and Cr (VI)^+^, respectively. Furthermore, gold (Au) nanoparticles were also decorated on Mg/Fe-LDO. The resulting Mg/Fe-LDO-AuNPs were found to possess excellent reducing properties and successfully converted 4-nitrophenol into 4-aminophenol in 5 min [44]. The Langmuir isotherm model was found to fit well compared to the Freundlich model while the pseudo-second-order model fitted well in kinetic studies of the adsorption process [44]. The designed material is cheaper, versatile, easy to prepare and has the potential for environmental applications in addition to water treatment. In another work, the mining wastewater containing heavy metal ions; Zn^2+^, Cd^2+^ and Pb^2+^ and an anion; SO_4_^2^^−^ [44] was successfully treated using a synthesized Mg/Al-LDHs, where SO4^2−^ from wastewater and heavy metal ions were concurrently intercalated and adsorbed, respectively on the LDHs. This strategy bypassed the multi-step process of synthesis work. The removal percentages were found to be 99% for Zn^2+^ and Pb^2+^ and 90% and 44% for Cd^2+^ and SO_4_^2−^. The process took about 2 h for the completion of the adsorption of metal ions and intercalation of SO_4_^2−^ [44]. Calcium-iron-based Ca/Fe-LDHs containing Cl^−^ and nitrate anion NO_3_^−^ were calcined (Ca/Fe-C-LDHs) and the resulting materials were used for the removal of arsenate. The adsorption capacities were found to be 156.0, 150.5 and 148.0 mg g^−1^ for the Cr^2+^, Cl^−^ and NO_3_, respectively. The isotherm study revealed that adsorption follows the Freundlich model and kinetic studies revealed that it follows pseudo-second-order [36]. The application of the calcined Ca/Fe-LDHs in real water samples was found to be able to purify, up to the WHO standards of drinking water with 99.80% removal of arsenate [36]. Water contamination by organic drugs also poses great environmental risks, such as the antibacterial drug, oxytetracycline which is used for the treatment of humans, animals, fish and plants. Mg/Al-LDHs were prepared and applied for the removal of oxytetracycline and some heavy metal ions; Cu^2+^, Ni^2+^, Co^2+^, Zn^2+^ and Fe^2+^ from water [58]. The removal percentage was found to be more than 99% within 120 min for all the metal ions and the organic drug molecule, oxytetracycline. The adsorption of all the metal ions and the organic drug followed the Langmuir isotherm model and the pseudo-first-order kinetics [58]. Nuclear technology is run between different countries for nuclear power; radioactive elements pose a great risk to the environment and human health. Therefore, the treatment of nuclear waste is of prime importance for environmental and human lives. Iodine is radio-active and poses a great risk to the environment. It exists in molecular iodine I_2_, HOI, and organic iodine. Iodine is widely used in many fields, such as chemical industries, research laboratories, medicine and agriculture, etc., [59]. There is an urgent need to develop a technique/material to capture radioactive iodine. For this, Cu/Bi LDHs containing CO_3_^−^ as the counter anion were synthesized by a simple co-precipitation method, resulting in a 3D hierarchical flower-like structure. The ZIF-67 linker has decorated the surface of the LDHs resulting in ZIF-67-Cu/Bi-LDHs [60]. Figure 2 shows a schematic illustration for the formation of the hierarchical flower-like structure of ZIF-67-Cu/Bi LDHs. They have successfully applied for the radioactive wastewater containing radioactive iodine treatment, resulting in the adsorption capacity of 139.98 mg g^−1^. The adsorption capacity was attributed to the special layered structure of the adsorbent in ZIF-67-Cu/Bi-LDHs and strong p-p an interaction between iodine and nitrogen of the imidazole ring of the ZIF67. The adsorption followed the Freundlich model isotherm and pseudo-second-order kinetics [60]. This material can be very useful for the treatment of nuclear wastewater which is a huge risk to aquatic life and human health [60].

## 7. Metal Oxides Nanoparticles

Latest studies have revealed that metal oxide nanoparticles have great potential for the removal of toxic metal ions wastewater [61]. Only a few metallic nanoparticles are analyzed for sorption due to their instability in agglomeration or separation. Furthermore, the separation of single metallic nanoparticles from wastewater is a difficult process [61]. Hence to stabilize their property and aggregate them in a simple way, they need to be functionalized. However, the field of nanoscience has introduced superior water purification techniques. The role of significant nanomaterials used in the water purification process includes the elimination of toxic metal ions and minute pollutants less than 300 nm and certain smart reagents with mechanical stability that can remove the toxic metal ions. Nanotechnology has been observed in more interest in the field of environmental application because of its higher surface area and tunable physicochemical properties. Metallic oxide semiconductors, such as titanium oxide and zinc oxide are capable of generating effectively for the removal of heavy metal water contamination. Then the separation of the multifunctional nanoparticles like ZnO is possible from the water systems after suspending for purification [62]. Another class of non-carbon nanomaterials is clay. Synthetic clay can be easily prepared using the bottom-up co-precipitation method and oxides of titanium and zinc oxide types can be decorated on it. The resulting materials can be used for heavy metal-contaminated water purification. These nanoclay-decorated TiO_2_ and ZnO have been found the best for the removal of a variety of contaminants in water and environmental remediation [63]. Nanoparticles of hybrid metallic Cu, Ag and Fe are used for antibacterial activity and de-contamination of toxic metal ions in the water system. In addition, water treatment of effluents from textile and tanning industries was leveraged on such nanoparticles or other multifunctional nanoparticles [64]. Another work on chromium ions elimination from wastewater was also used the adsorption method by titanium dioxide, activated carbon with metal fabrication, alumina, zeolites and in some studies, the reduction of Cr(III) is followed thereafter [65].

## 4. Magnetite Nanoparticles

**Figure 3:**
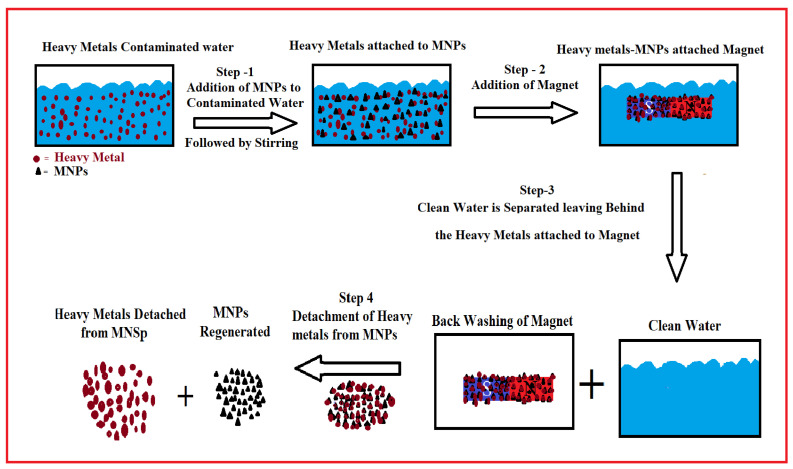
Schematic representation of the working steps for water cleaning using MNPs-RD.

Magnetite nanoparticles have been subjected to tremendous attention because of their unique-physicochemical properties, especially their high magnetization, unique electrical features, high surface area, small size and high adsorption capacity. Due to strong magnetic properties, they can easily be removed from water by placing a magnet and its surface can easily be functionalized with different surfactants. Some of the most commonly applied surfactants and polymeric coatings are polyvinylpyrrolidone (PVP), Polyethylene glycol (PEG), oleic acid, lauric acid, sulfonic acids and phosphonates, octanoic acid and chitosan, etc. Above all, these nanoparticles are cost-effective and easy to prepare at a large scale. These unique properties make them an ideal candidate for the treatment of wastewater. Iron oxide magnetite nanoparticles coated with polyvinylpyrrolidone (PVP–Fe_3_O_4_-NPs) have been successfully applied for the removal of heavy metal ions; Cd^2+^, Cr (VI), Ni^2+^ and Pb^2+^ from synthetic soft water and seawater, both in the absence and presence of fulvic acid. The PVP–Fe_3_O_4_ NPs were found to remove 100% of all metal ions at the concentration of 167 mg/L within 2 h and the kinetics was found to follow the pseudo-second-order. The material is useful for the removal of heavy metal ions under different environmental conditions; in the presence or absence of oil [48]. Iron oxide magnetite nanoparticles with a cubic crystalline structure stabilized them with three new surfactants namely 45OA, 55OA and 55+ with a size of below 20 nm were prepared [11]. The materials were applied for the removal of the Pb^2+^, Cd^2+^ and Zn^2+^ ions from water. The adsorption capacity of the magnetic material for all these three different ions was found to be near 40 mg/g and the adsorption was best fitted to the Langmuir isotherm [11]. A permanent magnetic Fe_3_O_4_ nanoparticles-based recovery device (MNPs-RD) that tended to purify water under continuous flow conditions was also prepared [66]. The resulting material can remove 94% arsenic. The MNPs-RD is an excellent, robust and versatile material that enabled large-scale treatment of heavy metal contaminated water [66]. Figure 3 shows a schematic flow MNPs-RD for the treatment of the contaminated water that has been resketched. Step 1 shows the contaminated water, step 2 shows the addition of MNPs powder to contaminated water followed by stirring resulting in the adsorption of contaminants on MNPs. The water resulting from step 2 passed through a chamber containing magnet resulting in attachment MNPs-heavy metals on the magnet. Followed by the separation of purified water (step 4) and backwashing of fluid (step 5). In step 6 MNPs are separated from the adsorbed contaminants. Bio-cryogel-coated MNPs (BCryo-MNPs) were synthesized and applied for the treatment of water containing Cr (VI), Pb^2+^, Cd^2+^ and Zn^2+^ heavy metal ions. They showed a high adsorption capacity of 2755, 2155, 3015 and 4100 mg/g for the Cr (VI), Pb^2+^, Cd^2+^ and Zn^2+^ ions, respectively. These nanoparticles have tremendous potential for the largescale production and industrial-scale treatment of heavy metal contaminated water [67]. Furthermore, Fe_3_O_4_ (MNPs) have been fabricated and coated with different materials and applied for environmental remediation and water purification. Few of them are briefly described here. Silica-coated magnetite-NH_2_ having a mesostructure has been applied for the removal of Cu^2+^ and the material showed an adsorption capacity of 0.5 mmol/g [68]. MNPs-nickel oxide-NPs, MNPs-montmorillonite, δ-FeOOH-coated γ-Fe2O3 MNPs, NPs-PEI have been applied for the removal of Cr (VI), with an adsorption capacity of 30, 15.3, 25.8 and 83.3 mg/g, respectively [15,25,69,70]. Hydrous-MNPs have been successfully applied for the removal of As^5+^ and Cr (VI), with an adsorption capacity of 8 mg/g for both of them. MNPs-Si-OH showed high removal efficiency of 97.34 and 90% for the Pb^2+^ and Hg^+^ ions, respectively [71]. Amino-modified Fe_3_O_4_ nanoparticles, MNPs-NH_2_ have been successfully applied for the removal of the Cu^2+^ and Cr (VI), ions with the maximum adsorption capacity of 12.43 and 11.24 mg/g, respectively [24,72].

## 5. Nanopolymers Composites (NPC)

There are various types of nanomaterials, which are polymerically designed. They have remarkably worked well in environmental remediation especially in water treatment. Polymeric nanomaterials are fabulous due to their high potential and specific functions and variety of dimensions. They are particularly suitable for the treatment of heavy metal contaminated water [73]. Different nano polymers e.g., sheets, needles and beads have different performances for removing heavy metal ions from water. The nano polymer usually worked as adsorbents in wastewater and was made from salts of metal nanoparticles like cellulose. Alginate and lignin just because of porosity of resins [74,75]. Magnetite NPs mixed with polystyrene showed the string adsorption function [76]. The presence of metal ions in polymers affects the removal of heavy metals from wastewater in an aqueous system. The formation of a tertiary structure around the nanocomposite enhances the function of the sorption of metal ions. The Fe_3_O_4_ magnetite NPs fabrication with polyethylene amine bonded between the layers of sodium montmorillonite at low pH 2 was researched as a good sorbent for Cr (VI) [77]. A powerful sorbent nanomaterial was formed by the combination of two polymers, acrylic acid mixed with grounded bentonite [78]. The lead, Nickel and Cadmium ions were found decontaminated. This process is good due to sorbent stabilization in their oxidation state. Nano derivatives of chitosan sorbents are analyzed for water contamination purification. Many reviews have covered chitosan nano derivatives with the best approval technically and scientifically [79]. Furthermore, the interaction of iron oxide magnetite-based nanocomposites makes for a more advanced material for wastewater purification with improved speed of sorption and can be reused and separated [66,77]. The usage of amine-fabrication magnet nanosorbents is best for toxic metal ions in wastewater. It can be summarized that chitosan polymers with magnetic nanoparticles of iron can be considered helpful for poisonous water purification. Commercially an advanced polymer nanocomposite of silicates has been created for heavy metal water purification and utilized in a low quantity and examined a prominent result. Porous polyurethane-keratin hybrid layers were used for the Cr (VI), removal in wastewater. Protein polymer was extracted from chicken feathers and mixed with polyurethane polymer to synthesize a hybrid [80,81]. Keratin was used by the adsorption method in which 38% of chromium ions were removed in an aqueous system. Keratin is considered a biosorbent for water treatment systems that can be used with a synthetic polymer to enhance the application of membranes for heavy metal contaminated water [81]. Poly pyrrole has been developed as one of the good polymer nanocomposites due to its performance for environmental remediation and stability. This material can be incorporated with any salt to increase the ion exchange property for toxic metal ions in wastewater. A novel organic-inorganic composite-poly pyrrole zirconium titanium phosphate has been prepared and analyzed using zirconium titanium phosphate, which is a modern material in ion-exchange material with good characteristics [82]. In general nanomaterials including traditional inorganic nano-sorbent and novel polymer supported composites are used to remove the heavy metal ions in wastewater treatment, due to their novel size- and shape-dependent properties, and gain the good to excellent removal efficiency [83].

## 6. Nanomembranes/Fibers

Nanoscience ideas go on the higher rank of performance for water purification by using nanomembranes which are having remarkable functionality due to reactivity, stability in catalytic action, and greater resistance for fouling. The focus is on the adsorption for this method and their positive percepts like high performance, impacts on heavy metal ions removal and less space occupied in a plant. While nanomembranes are also easy to synthesize they have a high financial cost [84]. Nanomembranes’ participation in removing heavy metal ions in an aqueous system shows the growth and functions of polyelectrolyte functionalized membranes. High-performance membranes were synthesized by grafting poly (acrylic acid) (PAA) and poly (iconic acid) (PIA) to cellulose nanofiber mats. Potential of PIA-modified membranes that exceeded 220 mg Cd/g by using Langmuir isotherm mode [84]. Furthermore, silver nanoparticles have been mixed with elongated ceramic filters, composed of wet clay-rich soil, which were powdered to smooth and various amounts and methods of coloring (dipping and painting). It was realized that nanoparticles of silver increased the performance of membrane used as filters and benefits to Escherichia coli removal about 100%. Detailed research on silver nanoparticles used in membranes enhances their role in waste water purification [85]. In this review paper, research and developments related to electrospun polymer nanofibers for metal ion removal are presented. The review highlights the emerging and increasing use of electrospun nanofibers for metal ion adsorption, especially for hazardous metals. Fundamental understanding of the electrospinning fabrication process, working parameters as they affect fiber morphologies, solvents and polymers used in electrospinning is discussed. This work summarizes the current status of the process of technology development and ideas in the research and use of electrospun nanofibers in heavy metal ion adsorption to address environmental problems from contaminated water. Zeolites and polysulfide membranes are utilized as an adsorbent in one research study for the decontamination of water containing Lead and Nickel ions. The Langmuir sorption isotherm model base calculation for the Pb^2+^ and Ni^2+^ were 682 and 122 mg/g of maximum adsorption capacity respectively [86]. While chromium and cobalt ion removal in aqueous solutions by using nanomaterials alumina fabricated with zeolites, were compared with sodium metal zeolite granules and the results were obtained; zeolites showed better and maximum efficiency in Cr (VI) 31.76%, Co^2+^ (17.2%) on fabricated alumina nanoparticles. Furthermore, studies of Nano zeolite with polyvinyl alcohol/sodium metal fabrication were tested in nickel and cadmium metal ions in a wastewater purification system. The comparative studies concluded with the high efficiency of polyvinyl acid over zeolite fibers for such metal ions. While the sorption capacity for cadmium was examined compared to nickel ions. The adsorption capacities for Cd^2+^ were observed to be much higher than Ni^2+^ [87]. The Adsorption characteristics of various non-carbon nanomaterials have been tabulated in Table 2. 

## 8. Metal-Organic Frameworks

Metal-organic frames (MOFs) are good catalytic nanomaterials for environmental remediation as well as other applications. MOFs have huge positive characteristics like greater surface area and superficial adjustment. MOFs have spongy-like surfaces with three-dimensional (3D) geometrical webs, which can be synthesized with metal ions and organic derivatives in a particular solvent. In the last few years, metallic organic frameworks have been utilized as reaction catalysts, absorption processes and membranes for filtration due to their high surface area and porosity. The special characteristics of MOFs have been taken as an interest for the application in gas technology, like gas adsorption, parting, gas packaging, gas detection, sensing and heterogeneous catalysis [88,89,90]. These multifunctional materials also have wide areas of application in gas sensors/separators, membranes, catalysis, etc. Air and water quality monitoring, food safety, medicine, defense are the important fields that require a novel technology for fabricating a device having desirable characteristics, such as low power requirements, selectivity, sensitivity and stability. MOFs are widely studied porous nanomaterials due to their exceptional degree of flexibility for both organic/inorganic ligands in the structure, which differentiate them from other organic nanostructures. The known strategies used in MOF synthesis, include different synthesis routes, precursor concentration, temperature and pressure and all of these factors play an important role in determining the resulting physicochemical properties. Cu-MOF has a high potential to be used for heavy metals elimination and higher efficiency than that of cobalt-based MOF (Co-MOF), suggesting that copper-MOF has a much-improved sorbent than Co-MOF. There was polystyrene (PS) nano-fibrous materials formed by the electrospinning method, which was then used as a template for thiol-functionalized mesoporous silica stuff. A macro-porous assembly, 3–10 μm in diameter, was observed in the polystyrene nanofibers’ structure which was diffused in the membrane. The optimized pH 5 for the sorption of Cu^2+^ was attained, with a sorption capacity of 11.33 mg/g, where Cu^2+^ ions were stickled on the surface of –SH linkages within the mesopores. The adsorbent dose was determined to be 0.32 mmol/g of the thiol group, and the adsorption efficiency (Cu^2+^/SH molar ratio) of the sorbent was suggested to be about 80%. The adsorptive capacity of CaCO_3_–pepsin for lead and copper ions at a pH 7 environment and the adsorptive potential of CaCO_3_–pepsin for Pb^2+^ and Cu^2+^ was determined to be 1167 and 611 mg/g, respectively, indicating the lead showed greater sorption. The solution yield of PbCO_3_ and CuCO_3_ was lower when compared to CaCO_3_, showing that Cu^2+^ and Pb^2+^ precipitate as their carbonates [91]. The adsorptive capabilities of CaCO_3_–maltose with hierarchical structure super-adsorbent, for Pb^2+^, Ni^2+^, Cu^2+^ and Cd^2+^ (as metal carbonate) were determined to be 3242.48, 769.23, 628.93 and 487.8 mg/g, respectively, whereas the adsorptive capability of CaCO_3_ for Pb^2+^ is 62.5 mg/g which indicates a good synergic effect between organic and inorganic moieties that exist in these compounds [92]. Based on the results, these organic-inorganic hierarchically structured compounds show super-sorbents properties. Owalude coworkers analyzed the sorption of Pb^2+^, Cd^2+^, Zn^2+^, Cu^2+^ and Hg^2+^ metals ions from their respective aqueous solution by treating formaldehyde (HCHO) and pyridine-fabricated with bean husks as the bio-sorbents. The adsorptive potential of the modified formaldehyde with bean husks (mg/g) was determined to be 5.0 mg/g; 3.63 mg/g; Zn^2+^ mg/g, 2.18 mg/g; 1.82 mg/g; 1.58 mg/g, while the pyridine-modified bean husk was analyzed to be 6.92; 3.63; 2.64; 2.48; 1.91 mg/g respectively [93]. Moreover, it was concluded while using isotherm models and q_max_ determined that the adsorptive potential of these metal ions were in the order of Pb^2+^ >Cd^2+^ >Zn^2+^ >Hg^2+^ >Cu^2+^ for formaldehyde-modified bean husks and Hg^2+^ >Cd^2+^ >Pb^2+^ >Zn^2+^ >Cu^2+^ for pyridine-modified bean husks [93].

**Photocatalysts: Metal-organic Frameworks (MOF):** With the fast growth of nanoscience and nanotechnology, the materials with nano-size have vast applications in the environmental area, where they cover the special space in wastewater treatment processes. The photo-catalysts nanomaterials have superior characteristics like high surface to volume ratio, easy modification, economically friendly, classical shape, composition and their actin in degradation reaction with transient metal ions or other elements and an outer alter oxidant, such as hydrogen peroxides. Furthermore, the process of intensification involves sono-hybrid advanced oxidation processes of sono-photocatalysis and a heterogeneous Fenton-like reaction for wastewater treatment [94]. Previous studies have shown that the nano-photo-catalysts, such as zirconium diamagnetic pentoxide, ZrFe_2_O_5_ having an α-Fe_2_O_3_ phase can act as a center of recombination for holes and electrons resulting in low photoactivity. However, this phase promotes a Fenton-like reaction in the presence of H_2_O_2_ leading to higher degradation. Therefore, the dual activities of photo and Fenton, ZrFe_2_O_5_, were found to be a better catalyst for hybrid advanced oxidation processes than other conventional photocatalysts [95]. On the other hand, the doping of transition metal ions into nano-photocatalysts helps to generate more (OH)-O-center radicals which attack the heavy metal ions and organic molecules adsorbed on the catalyst surface and enhance the degradation efficiency. In sono-hybrid advanced oxidation processes, such photocatalysts exhibit negative synergy as the intense shock waves generated due to the transient collapse of cavitation bubbles influence the desorption of organic molecules from the solid surfaces. As a result, low degradation efficiency was seen due to the reduction of interaction probability between radicals and organic molecules [94]. The dual photocatalysts like titanium dioxide and zeolite were studied for their work abilities on eliminating oxygen demand as chemical and sulfates from oil in water contaminants in the system. The dosage amount was optimized between (0.5–1.5 g/L), at contact time (15–45 min), agitation of (30–90 rpm) and their interactive effects were studied using the Box-Behnken design (BBD) of response surface methodology (RSM) [96]. An 18 watt of ultraviolet rays were introduced in the system for excitation of catalysts to initiate the reaction and end with the degradation with the removal of contaminants and TiO_2_ exhibited higher efficiency with a rate of 95% confidence level, the model’s predicted results were in good agreement with experimental data obtained [97]. There are various nano-photocatalysts like TiO_2_, GaP, WO_3_, ZnO, ZnS, CdS, etc., and have the super capability of light sensation and full potential of mineralization in water contaminants. Titanium dioxide is well known among them, with a photon energy of 400 nm and observed in high stability. There are numerous advanced oxidation processes applicable to wastewater treatment having toxic pollutants, which cannot be removed by other techniques. Advanced Oxidation Processes (AOPs) with H_2_O_2_/UV, O_3_/UV, O_3_/H_2_O_2_/UV and TiO_2_/UV start with the OH ion formation and can be derived for the oxidation mechanism through to complete mineralization. These radicals can destroy biologically refractory pollutants that are characterized by high chemical stability. Heavy metals (or metalloids) removal involves reduction reactions that produce elemental metals or metal ions at a lower oxidation state. The only exception is arsenic, which exists in the anionic form and needs oxidation to be converted to a high oxidation state [98]. Therefore, most of the AOPs are not useful processes as they cannot undergo a reduction reaction. However, TiO_2_/UV can endure both oxidation and reduction reactions and is applicable in all the heavy metal and metalloids removal/recovery processes [98]. Heterogeneous photocatalytic oxidation/reduction processes became popular among the AOPs, primarily because of the following reasons: (i) the processes can be carried out under ambient conditions (temperature and pressure), (ii) the oxidant is strong and less selective leading to complete mineralization, (iii) the processes do not consume any expensive oxidizing chemicals, (iv) it can undergo reduction reactions and (v) the photo-catalysts are less expensive, non-hazardous, stable and reusable.

**Silica Nanoparticles:** Silica nanoparticles are known for numerous empty pores, large surface area and high stability. Basically, there are three different kinds of silica nanoparticles, e.g., solid, nonporous and mesoporous [99]. Among these three classes, mesoporous silica nanoparticles have low polydispersibility, homogeneous structure, high surface area and have the ability to adsorb both by chemical and physical interactions [100]. Silica Nanoparticles (SNPs) have been widely exploited for the removal of heavy metal ions. Some of the most recent applications of SNPs in heavy metal contaminated water treatment are discussed here. Alswieleh et al. 2021 have designed mesoporous silica nanoparticles (MSN) with a very high surface area of 1048 m^2^g^−1^ with an average particles size of about 200 nm and functionalized them with amine, iminodiacetic acid, and glycine [99]. The designed functionalized MSN was applied for the removal of Cu^2+^, Co^2+^, Cr (VI), Pb^2+^ and Zn^2+^ metal ions. All these three functionalized MSN were found to be effective and removed about 95% of all the above-mentioned heavy metal ions at pH 3 [99]. In another study, Nasrin et al. 2021 fabricated the silica with sulfonic acid resulting in silica-sulfuric acid (SSA). The designed SSA was applied for the removal of heavy metal ions namely Cd^2+^, Ni^2+^, Mn^2+^, Pb^2+^ and Cu^2+^ from water. The removal efficiency of the designed SSA material was determined to be 80%, 69%, 88% 100%, and 99% for the Cd^2+^, Ni^2+^, Mn^2+^, Pb^2+^ and Cu^2+^ metal ions respectively at the optimized pH of 8 [101]. Hong et al. 2021 designed sulfhydryl functionalized mesoporous silica nanoparticles with a spherical shape and size range of 20–30nm with a very high surface area of 926 m^2^/g. They applied the designed nanomaterial material on the surface of the gold-silicon wafer. The designed wafer was capable of adsorbing the Pb^2+^, Cd^2+^ and Cr^3+^ with the highest adsorption of Cd^2+^ 28%, followed by 22% and 18% of Pb^2+^ and Cr^3+^ metal ions [102].Milton et al. 2020 designed the nano-silica hollow spheres (NSHS) having a crystal size of 39.5 nm. The designed nano-silica hollow spheres were able to adsorb 99.60% of Pb^2+^ metal ions at pH 5 [103]. Sofiya et al. 2017 designed amine and aromatic functionalized bilayer spherical silica nanoparticles and successfully applied them for the removal of Cu^2+^ and methylene blue dye. The removal of Cu^2+^ followed the mechanism of complexation with silica nanoparticles with a percentage removal of 80% in a 2.5 h short time [104]. The designed material was capable of removing 99.0 mg per gram of methylene blue. Furthermore, the Cu^2+^ silica complex was also found to show antibacterial properties [104]. Silica-based materials are one of the important classes of nanoparticles that are widely applied for water treatment and environmental remediation. They offer multiple advantages, such as being easy to synthesize, can easily be functionalized, can be prepared at a large scale, very economical. They also find their applications in biomedical sciences research.

## 9. Conclusions

Herein we have reviewed the advancement of non-carbon nanomaterials in heavy metal contaminated water treatment and environmental remediation. All these nanomaterials; LDHs, metal oxide, magnetite particles, metal-organic framework, nanocomposite, silica-based nanoparticles and some polymeric systems, etc., showed excellent adsorption properties for environmental remediation because of their high surface area, small size and stability. The electrospun nano-membrane has been designed as an advanced material for removing heavy metal ions like nickel, cadmium, chromium and others by using cellulose and amine silicates analyzed effectively for the process. The lead and nickel can be also removed in an aqueous solution by using chitosan nano mats. The use of electrospun nanofibers in metal ion adsorption is emerging and comparatively innovative. Nanofiber materials hold great potential in advancing the growth of metallurgical technologies and progress for the separation of metallic ions from various sources. The physiochemical removal processes of adsorption use nanofibers due to their large surface area to volume ratio and the availability of a wide variety of chemical and morphological modification methods. These are promising materials with great potential to be widely applied in environmental and water remediation. 

## Figures and Tables

**Table 2:** Adsorption characteristics of various non-carbon nanomaterials.

Adsorbent	Metal Ions	Adsorption Capacity (mg/g)/Removal (%)	Best Fitted Isotherm Model	Best Fitted Kinetic order	Reference
Biochar (Mg/A-LDH-BC)	Pb^2+^ CrO_4_^2−^	591.2 330.8 mg/g	Langmuir	Pseudo-Second-Order	[33]
Mg/Fe-LDO	As^5+^ Cr (VI),	178.6 mg g^−1^ 148.7 mg g^−1^	Langmuir	Pseudo Second Order	[34]
Ca/Fe-C-LDHs-Cl^-^ Ca/Fe-C-LDHs-NO_3_^-^	As^5+^	150.5 mg g^−1^, 148.0 mg g^−1^	Freundlich	Pseudo Second Order	[26]
Mg/Al-LDHs-oxytetracycline	Cu^2+^, Ni^2+^, Co^2+^, Zn^2+^ and Fe^2+^	99% of 60 mg/L	Langmuir	Pseudo Second Order	[43]
ZIF-67-Cu/Bi-LDHs	I^−^	139.98 mg g^−1^.	Freundlich	Pseudo Second Order	[45]
PVP–Fe_3_O_4_-NPs	Cd2+, Cr (VI), Ni2+ and Pb2+	99% of 1 mg/L	--	Pseudo Second Order	[38]
MNPs-RD	As^5+^	94% of 500 mg/L	--	--	[46]

Note: In this table -- means authors have not conducted that study.

## Data Availability

The data presented in this study are available on request from the corresponding author.
